# Insects With Survival Kits for Desiccation Tolerance Under Extreme Water Deficits

**DOI:** 10.3389/fphys.2018.01843

**Published:** 2018-12-21

**Authors:** Leena Thorat, Bimalendu B. Nath

**Affiliations:** Stress Biology Research Laboratory, Department of Zoology, Savitribai Phule Pune University, Pune, India

**Keywords:** insect ecology, humidity, temperature, climate change, stress, desiccation tolerance, anhydrobiosis, adaptation

## Abstract

The year 2002 marked the tercentenary of Antonie van Leeuwenhoek’s discovery of desiccation tolerance in animals. This remarkable phenomenon to sustain ‘life’ in the absence of water can be revived upon return of hydrating conditions. Today, coping with climate change-related factors, especially temperature-humidity imbalance, is a global challenge. Under such adverse circumstances, desiccation tolerance remains a prime mechanism of several plants and a few animals to escape the hostile consequences of fluctuating hydroperiodicity patterns in their habitats. Among small animals, insects have demonstrated impressive resilience to dehydration and thrive under physiological water deficits without compromising on revival and survival upon rehydration. The focus of this review is to compile research insights on insect desiccation tolerance, gathered over the past several decades from numerous laboratories worldwide working on different insect groups. We provide a comparative overview of species-specific behavioral changes, adjustments in physiological biochemistry and cellular and molecular mechanisms as few of the noteworthy desiccation-responsive survival kits in insects. Finally, we highlight the role of insects as potential mechanistic models in tracking global warming which will form the basis for translational research to mitigate periods of climatic uncertainty predicted for the future.

## Introduction

Long-term drought conditions leading to physiological water deficits are a threat to the survival and distribution of all organisms. To this notion, what comes as a delightful surprise is the demonstration of water loss mediated resurrection of apparently ‘dead’ organisms (Keilin, 1959). Such organisms have a remarkable ability of desiccation tolerance whereby they sustain cellular integrity in the desiccated form by activating unique physiological mechanisms (Clegg, 2001). Interestingly, this phase is reversible upon rehydration causing the revival and resumption of active metabolism. At present, global concerns include the challenges associated in coping with climatic stressors, especially the fallout due to humidity-temperature imbalance (Bellard et al., 2012; Boggs, 2016). Under the global sustainable development agendas^1^, research priorities on “life on land” (item#15) and “climate action” (item#13) have warranted attention. Among small animals, insects have proved to be reliable biological systems to anticipate cause-and-effect relations of climate change stressors (Addo-Bediako et al., 2001; Hoffmann and Todgham, 2010).

This mini-review highlights the notable adaptive mechanisms employed by insects to evade dehydration bouts in their habitats. There have been a few reviews on similar topics (Watanabe, 2006; Cornette and Kikawada, 2011; Chown et al., 2011; Sogame and Kikawada, 2017); however, no recent competent review has emphasized on the profound diversity of hygropreference and associated strategies in insects. Most importantly, we discuss the desiccation tolerance profiles in insects irrespective of whether they possess a lower tolerance potential or are anhydrobiotic with a tolerance for severe water loss. These aspects have not been fully appreciated in the past, therefore, we aim to compile the diverse range of insect desiccation stress responses from a general perspective. Lastly, the present evaluation is by no means an exhaustive list of all desiccation tolerant insects; nonetheless, many case studies have been gathered within the ambit of insect water stress management.

## Dry but Not Dead

The documented history of desiccation tolerance dates back to 370 BC when Theophrastus described conditions necessary to store *‘dry seeds alive*’ (Leprince and Buitink, 2015). Later, Antonie van Leeuwenhoek described his amazement over the dry dust containing *‘tiny dry animalcules’* that came to life within a few hours after being rehydrated with water (Keilin, 1959). Little did Leeuwenhoek know that his meticulous observations would form the basis of the latent phases of life. To describe this phenomenon, Giard (1894) coined the term ‘anhydrobiosis,’ an extreme form of desiccation tolerance which in Greek implies *‘life without water.’* ‘Desiccation avoidance’ and ‘desiccation tolerance’ are distinguishable phenomena (Pallarés et al., 2016). The former refers to the maintenance of water uptake and/or minimization of body water loss (e.g., *Folsomia candida*, Collembola: Isotomidae) while the latter includes organisms that can afford loss of water and sustain a dry form without compromising on revival upon rehydration (e.g., all anhydrobiotes). The threshold for tolerance of water loss is highly species-specific and striking differences in desiccation tolerance strategies and traits in congeneric insect species have been linked with their geographic locations and the frequency and duration of drought exposure (Marron et al., 2003; Strachan et al., 2015). However, this is not true in all insects such as few heliconiine butterflies (Lepidoptera: Nymphalidae) (Mazer and Appe, 2001). Contrary to the rationale that desert insects can withstand higher water loss than mesic species, the aquatic beetle, *Peltodytes muticus* (Coleoptera: Haliplidae) is known for its highest tolerance in comparison to the desert spider beetle, *Mezium affine* (Coleoptera: Ptinidae) (Pallarés et al., 2016). Closely related *Drosophila* species (Diptera: Drosophilidae) have evolved different water balance mechanisms as demonstrated in *D. nepalensis* vs. *D. takahashii* and *D. immigrans* vs. *D. nasuta* (Parkash et al., 2012a,b).

**FIGURE 1:**
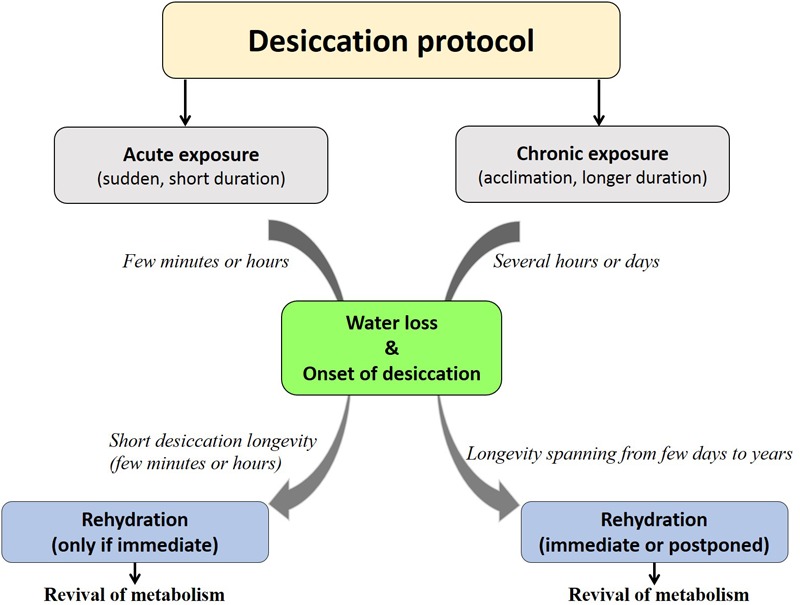
Fate of organisms under desiccation exposure. Acute desiccation permits shorter desiccation longevity (partial desiccation tolerance as seen in stenohygrobiotes) while chronic desiccation facilitates strategic competence to achieve higher desiccation longevity (extreme desiccation tolerance as seen in anhydrobiotes/euryhygrobiotes).

**FIGURE 2:**
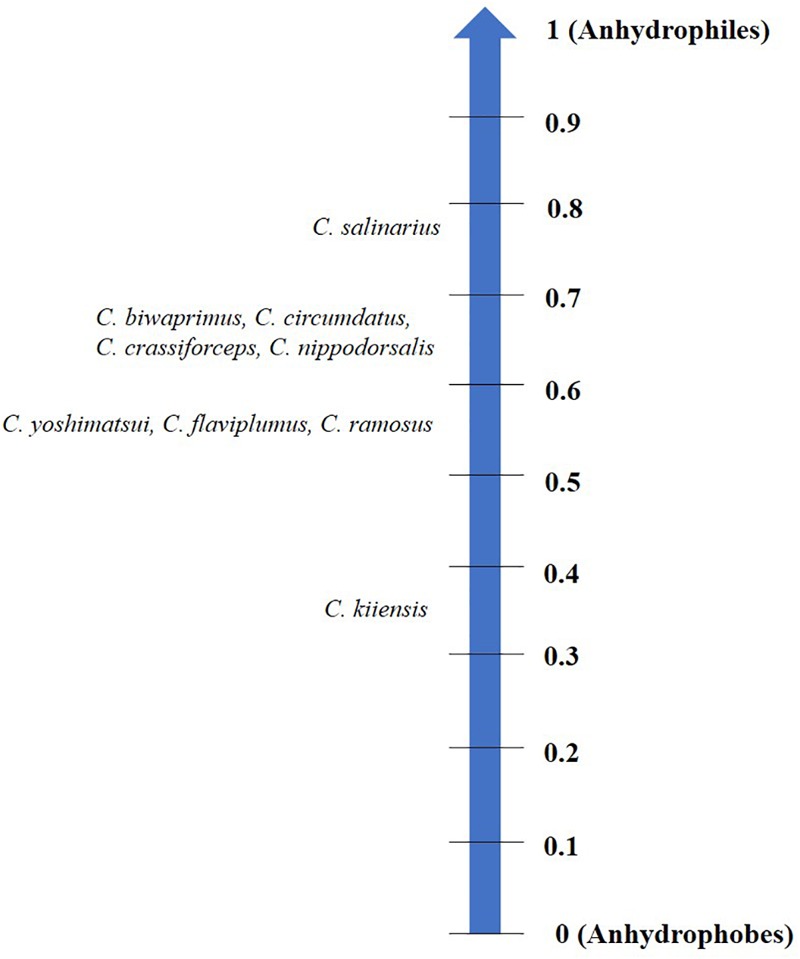
Desiccation tolerance index (DTi) scale categorizing nine oriental chironomids based on their threshold to tolerate water loss. Anhydrophobes lack desiccation tolerance while anhydrophiles are highly desiccation tolerant.

Each organism may have its specific threshold longevity in the dry state; however, desiccation tolerance by no means confers ‘immortality’ or infinite survival but is rather influenced by the mode of desiccation, storage temperature, humidity and oxygen content (Tunnacliffe and Lapinski, 2003; Suemoto et al., 2004; Thorat and Nath, 2016). Depending on these factors, organisms display varying longevities in the desiccated form that may range from 1 day to several years (Figure 1). Notwithstanding these variations and by virtue of qualitative considerations, all such organisms have been considered as desiccation tolerant (Watanabe, 2006). To the best of our knowledge, a numerical method devised for grouping prokaryotes based on their degree of desiccation tolerance, was the first attempt made by Hernández et al. (2009). A recent study in animals proposed the ‘desiccation tolerance index’ (DTi) as a quantitative measure of endurance to desiccation stress (Thorat and Nath, 2016). This mathematical tool is based on the desiccation tolerance in nine oriental *Chironomus* species (Diptera: Chironomidae) which indicate varying degrees of the tolerance threshold based on their ecological habitats (Figure 2).

## Anhydrobiosis: an Extreme Case of Desiccation Tolerance

Anhydrobiosis is characterized by extreme body water loss, generally over 95% (Benoit, 2010; Sogame and Kikawada, 2017). Thus, anhydrobiosis refers to complete desiccation, unlike desiccation tolerance, which refers to partial dehydration. In this context, we would like to introduce the term, ‘euryhygrobiote’ for such organisms that show a wide range of dehydration tolerance with a high anhydrobiotic potential. Conversely, we coin the term ‘stenohygrobiote’ for organisms that have a narrow dehydration tolerance range and can bear water loss only up to a certain limit. The extremophilic midges (Diptera: Chironomidae), *Polypedilum vanderplanki* (Hinton, 1951) and *Belgica antarctica* (Lopez-Martinez et al., 2009) are valuable models in understanding the gamut of molecular and biochemical signatures that render them anhydrobiotic. Anhydrobiotes can also be referred to as ‘anhydrophiles’ in comparison to ‘anhydrophobes,’ which lack desiccation tolerance. *P. vanderplanki*, the largest known anhydrobiotic eukaryote, endures water content as low as 3% through a gradual and optimized desiccation regime to sustain the dry state for 17 years until rehydration (Cornette and Kikawada, 2011). A new related species, *Polypedilum pembai* sp.n. also possesses anhydrobiotic potential and shares a few overlapping mechanisms with *P. vanderplanki* (Cornette et al., 2017). Recent work from our laboratory has demonstrated that the tropical midge, *Chironomus ramosus* and the fruit fly, *Drosophila melanogaster* possess a lower ability to tolerate water loss in comparison to the anhydrobiotic midges (Thorat et al., 2017) and are therefore stenohygrobiotic. Among invertebrates, other well-studied non-insect anhydrobiotes include brine shrimps, tardigrades, rotifers and nematodes (Tunnacliffe and Lapinski, 2003; Rebecchi, 2013). Interestingly, desiccation tolerance also confers cross tolerance to a variety of other stressors through multiple physiological defenses including physical and cellular protection *via* antioxidants, compatible solutes, proteins and DNA repair (Gusev et al., 2010b).

## Desiccation Tolerance Strategies in Insects

Environmental cues cause dormancy in insects, a phenomenon triggered by climatic signals including humidity, photoperiod, temperature, etc. (Diniz et al., 2017). Dormancy is further classified into diapause and quiescence. While diapause is a pre-programmed predictive strategy, quiescence is an immediate response to adverse environmental conditions (Denlinger, 1986; Danks, 2002). Aestivation, a form of consequential dormancy is the reason behind the aridity survival strategies of several insect species (Colvin, 1996; Benoit and Denlinger, 2007; North and Godfray, 2018). Anhydrobiosis (ametabolism) is an adaptation against physiological water stress, whereas dormancy is characterized by interrupted or reduced metabolic and hormonal activities (hypometabolism) in response to environmental cues (Watanabe, 2006).

While external milieu trigger desiccation stress responses, interoception is central to tolerance, survival and propagation of species. Below, we discuss a few of the striking and widely established strategies that constitute part of the desiccation tolerance approach of insects (Table 1).

**Table 1:** List of representative desiccation tolerant insects from different orders.

Order	Species	Life stage	Natural habitat	Reference
Collembola	*Folsomia candida*	Egg, larva, adult	Moist soil and sediments, leaf litter and decaying material	Sjursen et al., 2001; Holmstrup et al., 2002
	*Cryptopygus antarcticus, Friesea grisea*	Larva, adult	Moist Antarctic habitats	Alvarez et al., 1999; Hayward et al., 2004b; Elnitsky et al., 2008
	*Protaphorura tricampata Adult*		Meadows, mostly seashores	Holmstrup and Bayley, 2013
Ephemeroptera	*Cloeodes hydation*	Larva	Pools, ephemeral, rain-filled rock pools and springs	Nolte et al., 1996
Odonata	*Libellula depressa*	Larva	Still-water lakes and ponds (newly created ponds and well- vegetated ponds)	Rebora et al., 2007
Orthoptera	*Acheta domesticus*	Adult	Woodlands, caves, pastures, damp and soggy areas	McCluney and Date, 2008
	*Dianemobius nigrofasciatus*	Egg	Caves, fields, meadows, forests, grasslands, marshes and swamps.	Goto et al., 2008
	*Oedaleus senegalensis*	Egg, adult	Dry areas, annual grass communities, sandy soils	Colvin, 1996; Idrissa et al., 2008
Dictyoptera	*Periplaneta americana, Blattella germanica*	Nymph, adult	Humid spaces, cracks and crevices of porches, residential areas, temperate and tropical biomes, grasslands, rainforests and urban environments	Treherne and Willmer, 1975; Dambach and Goehlen, 1999
Phasmida	*Carausius morosus*	Egg, larva	Tropical forests, bushes and trees, garden plants, natural vegetation	Tichy, 1979
Plecoptera	*Protonemura intricate*,	Egg, larva	Freshwater, terrestrial and shredders of decayed tree leaves	Harper and Hynes, 1970; Marten and Zwick, 1989; Lancaster et al., 2010
Isoptera	*Macrotermes carbonarius, M. gilvus*	Adult	Terrestrial, subterranean	Hu et al., 2012
	*Coptotermes formosanus, Neotermes jouteli, Cryptotermes cavifrons, C. brevis*	Adult	Terrestrial, subterranean, dampwood	Zukowski and Su, 2017
Dermaptera	*Labidura riparia*	Nymph, larva, adult	Terrestrial, dark and moist environments, cultured and uncultured farmlands, woodlands, margins of ponds and lakes	Kharboutli and Mack, 1993
Hemiptera	*Cryptotympana facialis, Graptopsaltria nigrofuscata*	Nymph	Terrestrial, urban environments	Moriyama and Numata, 2010, 2011
	*Cimex lectularius*	Adult	Obligate blood feeders on humans	Benoit et al., 2007a
Trichoptera	*Lectrides varians*	Larva, pupa	Benthic, temperate lakes, streams, and ponds. Adults are terrestrial	Wickson et al., 2012
Lepidoptera	*Chlosyne lacinia*	Egg, Larva	Desert hills and woodlands	Clark and Faeth, 1998
	*Pieris brassicae, Aglais io, Heliconius charithonia*	Larva, Adult	Farms, tree trunks, walls and fences, in the vicinity of cruciferous plants	Willmer, 1980; Mazer and Appe, 2001
	*Manduca sexta*	Egg, larva	Facultative specialists on tobacco host plants	Rowley and Hanson, 2007; Davies et al., 2013
Hymenoptera	*Ceratosolen galili, Ceratosolen arabicus*	Adult	Terrestrial on host fig trees	Warren et al., 2010
	*Apis mellifera*	Adult	Temperate, tropical deserts, dunes, savannas, grasslands, swamps, urban and agricultural areas.	Atmowidjojo et al., 1997
Diptera	*Aedes albopictus, Culex pipiens, Anopheles gambiae*	Egg, Larva, Adult	Egg, larva and pupa are aquatic (freshwater), adults are terrestrial	Sota and Mogi, 1992; Alto and Juliano, 2001; Hidalgo et al., 2014; Wadaka et al., 2016; Diniz et al., 2017
	*P. vanderplankii, P. pembai, B. antarctica, C. ramosus, C. kiiensis, C. crassiforceps, C. nippodorsalis, C. biwaprimus, C. flaviplumus, C. salinarius, C. circumdatus, C. yoshimatsui*	Larva	Diverse aquatic habitats- African rock pools, rock pools of Malawi, Terrestrial Antarctic environments, tropical freshwater lakes and rivers, eutrophic lakes, rivers, ponds, artificial reservoirs and paddy fields	Suemoto et al., 2004; Benoit and Denlinger, 2007; Nakahara et al., 2008; Thorat and Nath, 2016; Thorat et al., 2017
	*Ceratitis capitate, C. cosyra, C. rosa, Bactrocera dorsalis*	Larva, adult	Fruit crop pest	Xie and Zhang, 2007; Weldon et al., 2016
	*Drosophila immigrans, D. pseudoobscura, D. hydei, D. mojavensis, D. birchii, D. nigrospiracula, D. nepalensis, D. takahashii, D. immigrans, D. nasuta, D. melanogaster, D. simulans*	Larva, adult	Deserts, tropical rainforest, cities, swamps, alpine zones, on decaying plant and fungal material	Hoffmann and Parsons, 1989; Davidson, 1990; Gibbs and Matzkin, 2001; Marron et al., 2003; Hoffmann et al., 2003; Bazinet et al., 2010; Thorat et al., 2012; Parkash et al., 2012a,b; Davies et al., 2013; Thorat et al., 2016b; Ferveur et al., 2018
	*Sarcophaga bullata*	Larva	Rural and urban environments, commonly found in houses and indoor dwellings	Yoder et al., 2006
Coleoptera	*Acanthoscelides obtectus*	Egg	Granivore, infesting seeds or beans and living inside them	Biemont et al., 1981
	*Longitarsus bethae*	Egg	Soil-dwelling, root-feeding on the host plant, *Lantana camara*	Simelane, 2007
	*Peltodytes muticus, Mezium affine, Enochrus halophilus, E. politus, E. bicolor, E. jesusarribasi*	Adult	Aquatic, temporary-lentic or intermittent-lotic water bodies, deserts	Arlian and Staiger, 1979; Pallarés et al., 2016
Siphonaptera	*Spilopsyllus cuniculi*	Egg, larva, pupa, adult	External parasite of rabbits	Cooke and Skewes, 1988
	*Ctenocephalides felis*	Egg, larva, pupa, adult	External parasite of cats	Silverman and Rust, 1983

### Behavior and Ecology

Hygrosensing abilities and behavioral responses suggest an evolutionary strategy for coping with water loss in insects (Chown et al., 2011). For instance, cockroaches show aggregation in order to control the water loss rate per individual (Dambach and Goehlen, 1999). Similar observations in *Chironomus* larvae indicate a ‘clumping’ behavior, forming a single bunch to reduce evaporative body water loss (Thorat and Nath, unpublished). Some beetles exhibit bimodal activity patterns in order to escape the hottest hours of the day whereas others display fog-basking for moisture absorption from the surroundings (Bedick et al., 2006; Chown et al., 2011). Other striking evidences for aridity protection, come from niche construction behaviors such as the housing nests of chironomid midges, termite nests, domiciles of some thrips and insect galls (Kikawada et al., 2005; Gilberta, 2014; Zukowski and Su, 2017; Thorat and Nath, 2018). The cuticle is the first portal of water loss in insects and the differential desiccation tolerance patterns in *C. ramosus* vs. *D. melanogaster* and *P. vanderplanki* vs. *Paraborniella tonnoiri* (Diptera: Chironomidae) have been attributed to striking differences in their cuticular thickness (Nakahara et al., 2008; Thorat et al., 2017). Furthermore, in some insects, restructuring of the cuticle and morphological changes in spiracular features are crucial to minimize water loss. Such restructuring mechanisms are important because water is mainly lost passively and/or actively throughout spiracular respiration and cuticular transpiration (Hadley, 1994; Benoit and Denlinger, 2007; Benoit, 2010; Bazinet et al., 2010; Wadaka et al., 2016; Hidalgo et al., 2018; Ferveur et al., 2018). Other behavioral traits for desiccation protection such as the arrangement of egg laying (layering and density) in the nymphalid butterfly, *Chlosyne lacinia* (Lepidoptera: Nymphalidae), increases desiccation survival chances of eggs (Clark and Faeth, 1998).

### Development and Hormonal Regulation

Our current understanding on the desiccation-mediated developmental consequences in insects is rather fragmented. In the case of the oriental fruit fly, *Bactrocera dorsalis* (Diptera: Tephritidae), desiccation does not exert significant effects on the average eclosion time (Xie and Zhang, 2007). In *C. ramosus and D. melanogaster*, modulations in 20-hydroxyecdysone affect recovery patterns and are linked with the desiccation-mediated delay in metamorphosis (Thorat and Nath, 2015; Thorat et al., 2016b). Interestingly, in *D. melanogaster*, despite the developmental heterochrony, the overall duration of postembryonic development of the life cycle remains almost unaltered. This is reminiscent of Waddington’s ‘canalization’ as an adaptive buffer to adjust their life histories around optimal seasonal conditions (Thorat et al., 2016b). Life cycle and aging in desiccation tolerant animals has been categorized into three hypothetical models, the first, known as the ‘Sleeping Beauty’ model, implies that organisms totally disregard the entire time spent in the dry state, the second model considers that organisms register partial discount of the time spent in the dry state and the third model, whereby organisms record the exact time spent in the dry state, exhibiting non-extended longevity. *D. melanogaster* follows the Sleeping Beauty model similar to the non-insect anhydrobiotic tardigrade, *Milnesium tardigradum* (Schill, 2010; Thorat et al., 2016b). Variations in insect hormonal titres are key players in synchronizing developmental changes in order to handle ecological ramifications of stressful environments such as hypoxia, high temperatures, starvation and sleep deprivation; however, investigations in the context of desiccation stress are warranted.

### Physiological Biochemistry

A longstanding biochemical adjustment of survival under dry conditions, is the ability of desiccation-responsive synthesis and accumulation of biomolecules including trehalose, mannitol, glycerol, Heat-Shock (HS) and Late Embryonic Abundant (LEA) proteins, proline, glycine-betaine, gamma aminobutyric acid, alanine, and glucosamine (Crowe and Madin, 1974; Tunnacliffe and Lapinski, 2003; Yoder et al., 2006; Kikawada et al., 2008; Philip et al., 2008; Benoit et al., 2009; Mitsumasu et al., 2010; Thorat et al., 2012; Hidalgo et al., 2014; Shukla et al., 2015, 2016, 2018; Yoshida et al., 2016; Thorat et al., 2017; Mazin et al., 2018). These compatible solutes not only offer protection to the drying tissues but also trigger various signaling responses during recovery. Although trehalose was considered indispensable for desiccation tolerance, recent compelling evidences have affirmed that trehalose accumulation may be completely absent in some organisms in which the desiccation protective role is taken up by other biomolecules (Tunnacliffe et al., 2005; Thorat et al., 2017). Differential physiological mechanisms involving carbohydrates, lipids and proteins are known to contribute to the invasive potential of three related *Ceratitis* fly species (Diptera: Tephritidae) under episodic dehydration (Weldon et al., 2016). Osmoregulatory mechanisms in lepidopteran species have demonstrated the homeostatic control to readjust hemolymph osmolality triggered by body water loss (Willmer, 1980). Interestingly, eggs of *Acanthoscelides obtectus* (Coleoptera: Bruchidae) show water loss coping mechanisms that enhance egg tolerance and survival (Biemont et al., 1981). In the case of the flea beetle, *Longitarsus bethae* (Chrysomelidae: Alticinae), while low relative humidity has no influence on oviposition, aridity beyond a critical point is lethal for the eggs (Simelane, 2007). In contrast, egg desiccation did not affect embryo survival in xeric and mesic populations of the tobacco hawk moth, *Manduca sexta* (Lepidoptera: Sphingidae) (Potter and Woods, 2012).

### Antioxidant Defense

Ionic imbalance and changes in osmolarity as a result of cellular water loss leads to the generation of reactive oxygen species (ROS) that are known to damage cellular macromolecules (Alpert, 2005; Benoit and Lopez-Martinez, 2012). Rebecchi (2013) has provided an excellent overview of the whole repertoire of antioxidant defenses under desiccation-responsive oxidative stress management in animals. *P.* vanderplanki shows the presence of both mitochondrial and cytosolic/extracellular superoxide dismutases (SODs) and abundant glutathione peroxidase and mitochondrial thioredoxin (Cornette et al., 2016; Nesmelov et al., 2016). Furthermore, genes that encode core components of enzymatic antioxidants in *P. nubifer* are similar to those in insects. However, in *P. vanderplanki* several groups of antioxidant genes have expanded (Gusev et al., 2014). In contrast, SOD serves as the major antioxidant in *B. antarctica* (Benoit and Lopez-Martinez, 2012). Recently, the role of unconventional antioxidant molecules such as trehalose, proline, polyamines and polyoils has gained attention (Goyal et al., 2004; Schill et al., 2009; Benoit and Lopez-Martinez, 2012). Trehalose, in particular, has been confirmed for its ROS-scavenging ability in SOD-deficient yeast cells and plants (Kranner and Birtič, 2005; França et al., 2007). Using the advantage of molecular genetic tools in *Drosophila* and a simple, non-invasive method of whole larval real-time imaging, Thorat et al. (2016a) have demonstrated for the first time that during desiccation, trehalose in collaboration with SOD is involved in the maintenance of redox homeostasis in insects.

### Molecular and Evolutionary Biology

Cellular decline in water levels serves as a cue to elicit defensive-responses of molecular indicators. Among the molecular responses mediated *via* proteins, Hsps, namely, smHsp, Hsp70 and Hsp90 have been linked with desiccation survival in insects (Tammariello et al., 1999; Sjursen et al., 2001; Hayward et al., 2004a; Benoit et al., 2009; Benoit, 2010). LEA proteins are another group of upregulated molecules that act as molecular shields to protect other proteins and bio-membranes against aggregation and denaturation resulting from drying (Goyal et al., 2005; Sogame and Kikawada, 2017). Interestingly, however, *B. antarctica* lacks genes encoding LEA proteins and Hsps are apparently not involved in conferring desiccation tolerance (Philip et al., 2008). Instead, metabolite synthesis and membrane phospholipids, distinct contractile and cytoskeletal protein patterns and aquaporins are among the key players essential for successful anhydrobiosis in the Antarctic midge (Benoit et al., 2007b; Michaud et al., 2008; Li et al., 2009; Teets et al., 2012; Kelley et al., 2014). In addition, desiccation response was shown to upregulate ‘*Frost*,’ ‘*Desi*’ and ‘*smp-30*’ genes whereas ‘*Desat2*’ was downregulated during post-desiccation recovery (Sinclair et al., 2007; Kawano et al., 2010). Metabolic fingerprint comparisons in mosquitoes have highlighted specific metabolic alterations, enabling them to survive seasonal aridity (Hidalgo et al., 2015). Diapause in *Aedes albopictus* (Diptera: Culicidae) promotes desiccation survival by overexpression of a transcript involved in lipid storage with a concomitant increase in hydrocarbon levels (Diniz et al., 2017). Seminal contributions from Davies et al. (2014) have deepened our understanding on the neuroendocrine regulation of salt and water balance in insects (Luan et al., 2015). Recently, the importance of capa neuropeptides as anti-diuretic hormones have been identified in *D. melanogaster* and is postulated to be a part of desiccation tolerance mechanisms in other insects as well (Davies et al., 2013; Terhzaz et al., 2015).

## Conclusion

Adaptive mechanisms vary among organisms based on their ecological and evolutionary background. Thus, stress tolerance physiology is bound to vary even among closely related species and therefore cannot be generalized. In addition, variations in desiccation tolerance physiology is often a result of the desiccation protocols (acute/chronic) employed. It might therefore be possible to judge the desiccation tolerance or anhydrobiotic potential of organisms in the true sense, only when they are studied under a common denominator of reproducible protocols. Nature has a vast array of tactics to safeguard its biodiversity and therefore, exploration of other aridity-induced mechanisms in known and unknown desiccation tolerant organisms will give way to our holistic understanding of the diversity in tolerance patterns from an evolutionary, ecological, physiological, cellular and molecular perspective. As reviewed here, although several molecular and biochemical underpinnings of desiccation tolerance in insects are thoroughly studied and well-established, an understanding of some other basic mechanisms remain elusive. For instance, there is a lack of information on the status of the immune responses elicited during desiccation survival. Another neglected area is the understanding of the neuronal basis governing recovery from desiccation that leads to the reactivation of coordinated sensory circuits. As an example, Pflüger and colleagues have determined the role of insect neurotransmitters in modulating multiple physiological and behavioral processes and have emphasized the involvement of biogenic amines under heat, mechanical stress, starvation and chemicals in insects (Verlinden et al., 2010). Similar studies on physiological water deficits in insects can hold great promise for translational research.

The role of insects as reliable mechanistic models presents endless research possibilities for the prediction of the consequences of climate change. The extreme desiccation tolerance of *P. vanderplanki* has been exploited as a prototype insect system for investigating the influence of spaceflight environments on life processes (Gusev et al., 2010a). Furthermore, the knowledge of insect desiccation biology offers ample ideas for exciting biomedical and pharmaceutical applications, e.g., anhydrobiotic engineering that targets at improving desiccation tolerance of desiccation-sensitive species, including humans (de Castro et al., 2000; Watanabe et al., 2016). These and many other applications that might have been previously viewed as science fiction, are now possible because of our knowledge of insect responses to water scarcity. Thus, research in desiccation stress response biology has come a long way from curiosity-driven explorations to present day technology-driven applications. Therefore, we hope that this review will trigger impetus for the development of methods and technology to mitigate the consequences of climate change in human and non-human biota.

## Author Contributions

LT and BN designed the review layout. LT prepared the manuscript draft, table, and figures. BN revised the manuscript with critical inputs. BN and LT approved the final version of the manuscript.

## Conflict of Interest Statement

The authors declare that the research was conducted in the absence of any commercial or financial relationships that could be construed as a potential conflict of interest.

## Footnotes

1 https://www.un.org/sustainabledevelopment/sustainable-development-goals/

## References

[B1] Addo-Bediako A. Chown S. L. Gaston K. J. (2001). Revisiting water loss in insects: a large scale view. *J. Insect Physiol.* 47 1377–1388. 10.1016/S0022-1910(01)00128-7 12770144

[B2] Alpert P. (2005). The limits and frontiers of desiccation-tolerant life. *Integr. Comp. Biol.* 45 685–695. 10.1093/icb/45.5.685 21676818

[B3] Alto B. W. Juliano S. A. (2001). Temperature effects on the dynamics of *Aedes albopictus* (Diptera: Culicidae) populations in the laboratory. *J. Med. Entomol.* 38 548–556. 10.1603/0022-2585-38.4.548 11476335 PMC2579928

[B4] Alvarez T. Frampton G. K. Goulson D. (1999). The effects of drought upon epigeal collembola from arable soils. *Agr. For. Entomol.* 1 243–248. 10.1046/j.1461-9563.1999.00032.x

[B5] Arlian L. Staiger T. (1979). Water balance in the semiaquatic beetle, *Peltodytes muticus*. *Comp. Biochem. Physiol. Part A Mol. Integr. Physiol.* 62A 1041–1047. 10.1016/0300-9629(79)90047-1

[B6] Atmowidjojo A. H. Wheeler D. E. Erickson E. H. Cohen A. C. (1997). Temperature tolerance and water balance in feral and domestic honey bees, *Apis mellifera* L. *Comp. Biochem. Physiol. Part A Physiol.* 118 1399–1403. 10.1016/S0300-9629(97)00031-5

[B7] Bazinet A. L. Marshall K. E. MacMillan H. A. Williams C. M. Sinclair B. J. (2010). Rapid changes in desiccation resistance in *Drosophila melanogaster* are facilitated by changes in cuticular permeability. *J. Insect Physiol.* 56 2006–2012. 10.1016/j.jinsphys.2010.09.002 20863831

[B8] Bedick J. C. Wyatt W. H. Albrecht M. C. (2006). High water-loss rates and rapid dehydration in the burying beetle, *Nicrophorus marginatus*. *Physiol. Entomol.* 31 23–29. 10.1111/j.1365-3032.2005.00477.x

[B9] Bellard C. Bertelsmeier C. Leadley P. Thuiller W. Courchamp F. (2012). Impacts of climate change on the future of biodiversity. *Ecol. Lett.* 15 365–377. 10.1111/j.1461-0248.2011.01736.x 22257223 PMC3880584

[B10] Benoit J. B. (2010). “Water management by dormant insects: comparisons between dehydration resistance during summer aestivation and winter diapause and aestivation,” in *Progress in Molecular and Subcellular Biology* eds Arturo Navas C. Carvalho J. (Berlin: Springer). 10.1007/978-3-642-02421-4_10 20069411

[B11] Benoit J. B. Denlinger D. L. (2007). Suppression of water loss during adult diapause in the northern house mosquito, *Culex pipiens*. *J. Exp. Biol.* 210 217–226. 10.1242/jeb.02630 17210959

[B12] Benoit J. B. Lopez-Martinez G. (2012). “Role of conventional and unconventional stress proteins during the response of insects to traumatic environmental conditions,” in *Hemolymph Proteins and Functional Peptides: Recent Advances in Insects and Other Arthropods* eds Tufail M. Takeda M. (Oak Park, IL: Bentham Science) 128–160.

[B13] Benoit J. B. Del Grosso N. A. Yoder J. A. Denlinger D. L. (2007a). Resistance to dehydration between bouts of blood feeding in the bed bug, *Cimex lectularius*, is enhanced by water conservation, aggregation, and quiescence. *Am. J. Trop. Med. Hyg.* 76 987–993. 17488928

[B14] Benoit J. B. Lopez-Martinez G. Michaud M. R. Elnitsky M. A. Lee R. E. Jr. Denlinger D. L. (2007b). Mechanisms to reduce dehydration stress in larvae of the Antarctic midge *Belgica antarctica*. *J. Insect Physiol.* 53 656–667. 10.1016/j.jinsphys.2007.04.006 17543329

[B15] Benoit J. B. Lopez-Martinez G. Phillips Z. P. Patrick K. R. Denlinger D. L. (2009). Heat shock proteins contribute to mosquito dehydration tolerance. *J. Insect Physiol.* 56 151–156. 10.1016/j.jinsphys.2009.09.012 19782687 PMC2861860

[B16] Biemont J. C. Chauvin G. Hamon C. (1981). Ultrastructure and resistance to water loss in eggs of *Acanthoscelides obtectus* say (Coleoptera: Bruchidae). *J. Insect Physiol.* 27 667–679. 10.1016/0022-1910(81)90003-2

[B17] Boggs C. L. (2016). The fingerprints of global climate change on insect populations. *Curr. Opin. Insect Sci.* 17 69–73. 10.1016/j.cois.2016.07.004 27720076

[B18] Chown S. L. Sørense J. G. Terblanche J. S. (2011). Water loss in insects: an environmental change perspective. *J. Insect Physiol.* 57 1070–1084. 10.1016/j.jinsphys.2011.05.004 21640726

[B19] Clark B. R. Faeth S. H. (1998). The evolution of egg clustering in butterflies: a test of the egg desiccation hypothesis. *Evol. Ecol.* 12 543–552. 10.1023/A:1006504725592

[B20] Clegg J. (2001). Cryptobiosis- a peculiar state of biological organization. *Comp. Biochem. Physiol. Part B* 128 613–624. 10.1016/S1096-4959(01)00300-1 11290443

[B21] Colvin J. (1996). Diapause duration, survival in relation to desiccation and egg-pod morphology of the *Senegalese grasshopper, Oedaleus senegalensis*. *Physiol. Entomol.* 21 173–178. 10.1111/j.1365-3032.1996.tb00852.x

[B22] Cooke B. D. Skewes M. K. (1988). The effects of temperature and humidity on the survival and development of the European rabbit flea, *Spilopsyllus cuniculi* (Dale). *Aust. J. Zool.* 36 649–659. 10.1071/ZO9880649

[B23] Cornette R. Kikawada T. (2011). The induction of anhydrobiosis in the sleeping chironomid: current status of our knowledge. *IUBMB Life* 63 419–429. 10.1002/iub.463 21547992

[B24] Cornette R. Kikawada T. Shagimardanova E. I. (2016). New antioxidant genes from an anhydrobiotic insect: unique structural features in functional motifs of thioredoxin. *Bionanoscience* 6 568–570. 10.1007/s12668-016-0278-x

[B25] Cornette R. Yamamoto N. Yamamoto M. Kobayashi T. Petrova N. A. Gusev O. (2017). A new anhydrobiotic midge from Malawi, *Polypedilum pembai* sp.n. (Diptera: Chironomidae), closely related to the desiccation tolerant midge, *Polypedilum vanderplanki* Hinton. *Sys. Entomol.* 42 814–825. 10.1111/syen.12248

[B26] Crowe J. H. Madin K. A. (1974). Anhydrobiosis in tardigrades and nematodes. *Trans. Am. Microsc. Soc.* 93 513–524. 10.2307/3225155

[B27] Dambach M. Goehlen B. (1999). Aggregation density and longevity correlate with humidity in first-instar nymphs of the cockroach (Blattella germanica L., Dictyoptera). *J. Insect Physiol.* 45 423–429. 10.1016/S0022-1910(98)00141-3 12770325

[B28] Danks H. V. (2002). The range of insect dormancy responses. *J. Entomol.* 99 127–142. 10.14411/eje.2002.021

[B29] Davidson J. K. (1990). Nonparallel geographic patterns for tolerance to cold and desiccation in *Drosophila melanogaster* and *Drosophila simulans*. *Aust. J. Zool.* 38 155–161. 10.1071/ZO9900155

[B30] Davies S. A. Cabrero P. Overend G. Aitchison L. Sebastian S. Terhzaz S. (2014). Cell sigalling mechanisms for insect stress tolerance. *J. Exp. Biol.* 217 119–128. 10.1242/jeb.090571 24353211

[B31] Davies S. A. Cabrero P. Povsic M. Johnston N. R. Terhzaz S. Dow J. A. (2013). Signaling by Drosophila capa neuropeptides. *Gen. Comp. Endocrinol.* 1 60–66. 10.1016/j.ygcen.2013.03.012 23557645

[B32] de Castro A. G. Bredholt H. Strøm A. R. Tunnacliffe A. (2000). Anhydrobiotic engineering of gram-negative bacteria. *Appl. Environ. Microbiol.* 66 4142–4144. 10.1128/AEM.66.9.4142-4144.2000 10966444 PMC92274

[B33] Denlinger D. L. (1986). Dormancy in tropical insects. *Ann. Rev. Entornol.* 31 239–264. 10.1146/annurev.en.31.010186.001323 3510585

[B34] Diniz D. F. A. de Albuquerque C. M. Oliva L. O. de Melo-Santos M. A. V. Ayres C. F. J. (2017). Diapause and quiescence: dormancy mechanisms that contribute to the geographical expansion of mosquitoes and their evolutionary success. *Parasit. Vectors* 10:310. 10.1186/s13071-017-2235-0 28651558 PMC5485599

[B35] Elnitsky M. A. Benoit J. B. Denlinger D. L. Lee R. E. Jr. (2008). Desiccation tolerance and drought acclimation in the *Antarctic collembolan Cryptopygus antarcticus*. *J. Insect Physiol.* 54 1432–1439. 10.1016/j.jinsphys.2008.08.004 18761345

[B36] Ferveur J.-F. Corto J. Rihani K. Cobb M. Everaerts C. (2018). Desiccation resistance: effect of cuticular hydrocarbons and water content in *Drosophila melanogaster* adults. *PeerJ.* 6:e4318. 10.7717/peerj.4318 29456884 PMC5813593

[B37] França M. B. Panek A. D. Eleutherio E. C. A. (2007). Oxidative stress and its effects during dehydration. *Comp. Biochem. Physiol. Part A Mol. Intg. Physiol.* 146 621–631. 10.1016/j.cbpa.2006.02.030 16580854

[B38] Giard A. (1894). L’anhydrobiose ou ralentissement des phénoménes vitaux. *C. R. Soc. Biol.* 46 497–500.

[B39] Gibbs A. G. Matzkin L. M. (2001). Evolution of water balance in the genus Drosophila. *J. Exp. Biol.* 204 2331–2338. 11507115 10.1242/jeb.204.13.2331

[B40] Gilberta J. D. J. (2014). Thrips domiciles protect larvae from desiccation in an arid environment. *Behav. Ecol.* 25 1338–1346. 10.1093/beheco/aru128 25419084 PMC4235581

[B41] Goto S. G. Doi K. Nakayama S. Numata H. (2008). Maternal control of cold and desiccation tolerance in eggs of the band-legged ground cricket *Dianemobius nigrofasciatus* in relation to embryonic diapause. *Entomol. Res.* 38 17–23. 10.1111/j.1748-5967.2008.00140.x

[B42] Goyal K. Browne J. A. Walton L. J. Pinelli C. Rastogi R. K. Burnell A. M. (2004). Molecular anhydrobiology: identifying molecules implicated in invertebrate anhydrobiosis. *Integr. Comp. Biol.* 45 702–709. 10.1093/icb/45.5.702 21676820

[B43] Goyal K. Walton L. J. Tunnacliffe A. (2005). LEA proteins prevent protein aggregation due to water stress. *Biochem. J.* 388 151–157. 10.1042/BJ20041931 15631617 PMC1186703

[B44] Gusev O. Nakahara Y. Sychev V. Levinskikh M. Novikova N. Alexeev V. (2010a). An anhydrobiotic insect, *Polypedilum vanderplanki* as a tool for astrobiology. *Space Utiliz. Res.* 24 306–309.

[B45] Gusev O. Nakahara Y. Vanyagina V. Malutina L. Cornette R. Sakashita T. (2010b). Anhydrobiosis-associated nuclear dna damage and repair in the sleeping chironomid: linkage with radioresistance. *PLoS One* 5:e14008. 10.1371/journal.pone.0014008 21103355 PMC2982815

[B46] Gusev O. Suetsugu Y. Cornette R. Kawashima T. Logacheva M. D. Kondrashov A. S. (2014). Comparative genome sequencing reveals genomic signature of extreme desiccation tolerance in the anhydrobiotic midge. *Nat. Commun.* 5:4784. 10.1038/ncomms5784 25216354 PMC4175575

[B47] Hadley N. F. (1994). *Water Relations of Terrestrial Arthropods.* San Diego: Academic Press.

[B48] Harper P. P. Hynes H. B. N. (1970). Diapause in the nymphs of Canadian winter stoneflies. *Ecology* 51 925–927. 10.2307/1933992

[B49] Hayward S. A. L. Rinehart J. P. Denlinger D. L. (2004a). Desiccation and rehydration elicit distinct heat shock protein transcript responses in flesh fly pupae. *J. Exp. Biol.* 207 963–997. 10.1242/jeb.00842 14766955

[B50] Hayward S. A. L. Worland M. R. Convey P. Bale J. S. (2004b). Habitat moisture availability and the local distribution of the antarctic collembola *Cryptopygus antarcticus* and *Friesea grisea*. *Soil Biol. Biochem.* 36 927–934. 10.1016/j.soilbio.2004.02.007

[B51] Hernández A. Zamora J. González N. Salazar E. Sánchez M. D. (2009). Anhydrobiosis quotient: a novel approach to evaluate stability in desiccated bacterial cells. *J. Appl. Microbiol.* 107 436–442. 10.1111/j.1365-2672.2009.04216.x 19291234

[B52] Hidalgo K. Montazeau C. Siaussat D. Braman V. Trabalon M. Simard F. (2018). Distinct physiological, biochemical and morphometric adjustments in the malaria vectors *Anopheles gambiae* and *An. coluzzii* as means to survive to dry season conditions in Burkina Faso. *J. Exp. Biol.* 21:jeb.174433. 10.1242/jeb.174433 29378815

[B53] Hidalgo K. Mouline K. Mamai W. Foucreau N. Dabiré K. R. Bouchereau A. (2014). Novel insights into the metabolic and biochemical underpinnings assisting dry-season survival in female malaria mosquitoes of the *Anopheles gambiae* complex. *J. Insect Physiol.* 70 102–116. 10.1016/j.jinsphys.2014.07.003 25083809

[B54] Hidalgo K. Mouline K. Mamai W. Foucreau N. Dabiré K. R. Bouchereau A. (2015). Combining two-dimensional gel electrophoresis and metabolomic data in support of dry-season survival in the two main species of the malarial mosquito *Anopheles gambiae*. *Data Brief* 5 255–268. 10.1016/j.dib.2015.08.031 26543889 PMC4589799

[B55] Hinton H. E. (1951). A new chironomid from Africa, the larva of which can be dehydrated without injury. *Proc. Zool. Soc. Lond.* 121 371–380. 10.1111/j.1096-3642.1951.tb00801.x

[B56] Hoffmann A. A. Hallas R. J. Dean J. A. Schiffer M. (2003). Low potential for climatic stress adaptation in a rainforest Drosophila species. *Science* 301 100–102. 10.1126/science.1084296 12843394

[B57] Hoffmann A. A. Parsons P. A. (1989). An integrated approach to environmental stress tolerance and life-history variation: desiccation tolerance in Drosophila. *Biol. J. Linn. Soc.* 37 117–136. 10.1111/j.1095-8312.1989.tb02098.x

[B58] Hoffmann G. E. Todgham A. E. (2010). Living in the now: physiological mechanisms to tolerate a rapidly changing environment. *Annu. Rev. Physiol.* 72 127–145. 10.1146/annurev-physiol-021909-135900 20148670

[B59] Holmstrup M. Bayley M. (2013). Protaphorura tricampata, a euedaphic and highly permeable springtail that can sustain activity by osmoregulation during extreme drought. *J. Insect Physiol.* 59 1104–1110. 10.1016/j.jinsphys.2013.08.015 24035747

[B60] Holmstrup M. Hedlund K. Boriss H. (2002). Drought acclimation and lipid composition in *Folsomia candida*: implications for cold shock, heat shock and acute desiccation stress. *J. Insect Physiol.* 48 961–970. 10.1016/S0022-1910(02)00175-0 12770043

[B61] Hu J. Neoh K. B. Appel A. G. Lee C. Y. (2012). Subterranean termite open-air foraging and tolerance to desiccation: comparative water relation of two sympatric Macrotermes spp. (Blattodea: Termitidae). *Comp. Biochem. Physiol. A Mol. Integr. Physiol.* 161 201–217. 10.1016/j.cbpa.2011.10.028 22085890

[B62] Idrissa M. Lecoq M. Kooyman C. (2008). Ecology and management of the *Senegalese grasshopper, Oedaleus senegalensis* (Krauss, 1877) (Orthoptera: Acrididae), in West Africa. review and prospects. *Ann. Soc. Entomol. France* 44 271–288. 10.1080/00379271.2008.10697563

[B63] Kawano T. Shimoda M. Matsumoto H. Ryuda M. Tsuzuki S. Hayakawa Y. (2010). Identification of a gene, Desiccate, contributing to desiccation resistance in *Drosophila melanogaster*. *J. Biol. Chem.* 285 38889–38897. 10.1074/jbc.M110.168864 20937803 PMC2998084

[B64] Keilin D. (1959). The problem of anabiosis or latent life: history and current concept. *Proc. R. Soc. Lond. B* 150 149–191. 10.1098/rspb.1959.0013 13633975

[B65] Kelley J. L. Peyton J. T. Fiston-Lavier A.-S. Teets N. M. Yee M.-C. Johnston J. S. (2014). Compact genome of the antarctic midge is likely an adaptation to an extreme environment. *Nat. Commun.* 5:4611. 10.1038/ncomms5611 25118180 PMC4164542

[B66] Kharboutli M. S. Mack T. P. (1993). Tolerance of the striped earwig (Dermaptera: Labiduridae) to hot and dry conditions. *Envtl. Entomol.* 22 663–668. 10.1093/ee/22.3.663

[B67] Kikawada T. Minakawa N. Watanabe M. Okuda T. (2005). Factors inducing successful anhydrobiosis in the African chironomid *Polypedilum vanderplanki*: significance of the larval tubular nest. *Integr. Comp. Biol.* 45 710–714. 10.1093/icb/45.5.710 21676821

[B68] Kikawada T. Saito A. Kanamori Y. Fujita M. Śnigórska K. Watanabe M. (2008). Dehydration-inducible changes in expression of two aquaporins in the sleeping chironomid, *Polypedilum vanderplanki*. *Biochim. Biophys. Acta* 1778 514–520. 10.1016/j.bbamem.2007.11.009 18082130

[B69] Kranner I. Birtič S. (2005). A modulating role for antioxidants in desiccation tolerance. *Integr. Comp. Biol.* 45 451–460. 10.1093/icb/45.5.734 21676824

[B70] Lancaster J. Downes B. J. Arnold A. (2010). Environmental constraints on oviposition limit egg supply of a stream insect at multiple scales. *Oecologia* 163 373–384. 10.1007/s00442-010-1565-9 20112109

[B71] Leprince O. Buitink J. (2015). Introduction to desiccation biology: from old borders to new frontiers. *Planta* 242 369–378. 10.1007/s00425-015-2357-6 26142353

[B72] Li A. Benoit J. B. Lopez-Martinez G. Elnitsky M. A. Lee R. E. Jr. Denlinger D. L. (2009). Distinct contractile and cytoskeletal protein patterns in the Antarctic midge are elicited by desiccation and rehydration. *Proteomics* 9 2788–2797. 10.1002/pmic.200800850 19415656

[B73] Lopez-Martinez G. Benoit J. B. Rinehart J. P. Elnitsky M. A. Lee R. E. Denlinger D. L. (2009). Dehydration, rehydration, and overhydration alter patterns of gene expression in the *Antarctic midge, Belgica antarctica*. *J. Comp. Physiol. B* 179 481–491. 10.1007/s00360-008-0334-0 19125254

[B74] Luan Z. Quigley C. Li H. (2015). The putative Na+/Cl- dependent neurotransmitter/osmolyte transporter inebriated in the Drosophila hindgut is essential for the maintenance of systemic water homeostasis. *Sci. Rep.* 5:7993. 10.1038/srep07993 25613130 PMC4303880

[B75] Marron M. T. Markow T. A. Kain K. J. Gibbs A. G. (2003). Effects of starvation and desiccation on energy metabolism in desert and mesic Drosophila. *J. Insect Physiol.* 49 261–270. 10.1016/S0022-1910(02)00287-1 12770001

[B76] Marten M. Zwick P. (1989). The temperature dependence of embryonic and larval development in Protonemura intricata (Plecoptera: Nemouridae). *Freshw. Biol.* 22 1–14. 10.1111/j.1365-2427.1989.tb01079.x

[B77] Mazer C. Appe A. (2001). Water loss and desiccation tolerances of longwing butterflies (Lepidoptera: Nymphalidae). *Environ. Entomol.* 30 631–636. 10.1603/0046-225X-30.4.631

[B78] Mazin P. V. Shagimardanova E. Kozlova O. Cherkasov A. Sutormin R. Stepanova V. V. (2018). Cooption of heat shock regulatory system for anhydrobiosis in the sleeping chironomid *Polypedilum vanderplanki*. *Proc. Natl. Acad. Sci. U.S.A.* 6 E2477–E2486. 10.1073/pnas.1719493115 29463761 PMC5877948

[B79] McCluney K. E. Date R. C. (2008). The effects of hydration on growth of the house cricket, *Acheta domesticus*. *J. Insect Sci.* 8:32. 10.1673/031.008.3201 20302456 PMC3061604

[B80] Michaud M. R. Benoit J. B. Lopez-Martinez G. Elnitsky M. A. Lee R. E. Denlinger D. L. (2008). Metabolomics reveals unique and shared metabolic changes in response to heat shock, freezing, and desiccation in the *Antarctic midge, Belgica antarctica*. *J. Insect Physiol.* 54 645–655. 10.1016/j.jinsphys.2008.01.003 18313070

[B81] Mitsumasu K. Kanamori Y. Fujita M. Iwata K. Tanaka D. Kikuta S. (2010). Enzymatic control of anhydrobiosis-related accumulation of trehalose in the sleeping chironomid, *Polypedilum vanderplanki*. *FEBS J.* 277 4215–4228. 10.1111/j.1742-4658.2010.07811.x 20825482 PMC3037560

[B82] Moriyama M. Numata H. (2010). Desiccation tolerance in fully developed embryos of two cicadas, *Cryptotympana facialis* and *Graptopsaltria nigrofuscata*. *Entomol. Sci.* 13 68–74. 10.1111/j.1479-8298.2010.00365.x

[B83] Moriyama M. Numata H. (2011). A cicada that ensures its fitness during climate warming by synchronizing its hatching time with the rainy season. *Zool. Sci.* 28 875–881. 10.2108/zsj.28.875 22132784

[B84] Nakahara Y. Watanabe M. Fujita A. Kanamori Y. Tanaka D. Iwata K. (2008). Effects of dehydration rate on physiological responses and survival after rehydration in larvae of the anhydrobiotic chironomid. *J. Insect Physiol.* 54 1220–1225. 10.1016/j.jinsphys.2008.05.007 18652833

[B85] Nesmelov A. A. Devatiyarov R. M. Voronina T. A. Kondratyeva S. A. Cherkasov A. V. Cornette R. (2016). New antioxidant genes from an anhydrobiotic insect: unique structural features in functional motifs of thioredoxin. *Bionanoscience* 6 568–570. 10.1007/s12668-016-0278-x

[B86] Nolte U. Tietböhl R. S. McCafferty W. P. (1996). A mayfly from tropical Brazil capable of tolerating short-term dehydration. *J. North Am. Benthol. Soc.* 15 87–94. 10.2307/1467434

[B87] North A. R. Godfray H. C. (2018). Modelling the persistence of mosquito vectors of malaria in Burkina Faso. *Malar. J.* 17:140. 10.1186/s12936-018-2288-3 29609598 PMC5879775

[B88] Pallarés S. Velasco J. Millán A. Bilton D. T. Arribas P. (2016). Aquatic insects dealing with dehydration: do desiccation resistance traits differ in species with contrasting habitat preferences? *PeerJ* 4:e2382. 10.7717/peerj.2382 27635346 PMC5012287

[B89] Parkash R. Aggarwal D. D. Ranga P. Singh D. (2012a). Divergent strategies for adaptation to desiccation stress in two Drosophila species of immigrans group. *J. Comp. Physiol. B* 182 751–769. 10.1007/s00360-012-0655-x 22407357

[B90] Parkash R. Ramniwas S. Kajla B. Aggarwal D. D. (2012b). Divergence of desiccation-related traits in two Drosophila species of the takahashii subgroup from the western Himalayas. *J. Exp. Biol.* 215 2181–2191. 10.1242/jeb.065730 22675178

[B91] Philip B. N. Yi S.-X. Elnitsky M. A. Lee R. E. Jr. (2008). Aquaporins play a role in desiccation and freeze tolerance in larvae of the goldenrod gall fly, *Eurosta solidaginis*. *J. Exp. Biol.* 211 1114–1119. 10.1242/jeb.016758 18344486

[B92] Potter K. A. Woods H. A. (2012). No evidence for the evolution of thermal or desiccation tolerance of eggs among populations of *Manduca sexta*. *Funct. Ecol.* 26 112–122. 10.1111/j.1365-2435.2011.01912.x

[B93] Rebecchi L. (2013). Dry up and survive: the role of antioxidant defences in anhydrobiotic organisms. *J. Limnol.* 72 62–72. 10.4081/jlimnol.2013.s1.e8

[B94] Rebora M. Piersanti S. Salerno G. Conti E. Gaino E. (2007). Water deprivation tolerance and humidity response in a larval dragonfly: a possible adaptation for survival in drying ponds. *Physiol. Entomol.* 32 121–126. 10.1111/j.1365-3032.2006.00553.x

[B95] Rowley M. Hanson F. (2007). Humidity detection and hygropreference behavior in larvae of the tobacco hornworm, *Manduca sexta*. *J. Insect Sci.* 7:39. 10.1673/031.007.3901 20302460 PMC2999434

[B96] Schill R. O. (2010). “Anhydrobiotic abilities of tardigrades,” in *Dormancy and Resistance in Harsh Environments* eds Lubzens E. Cerda J. Clark M. (Heidelberg: Springer-Verlag) 133–146.

[B97] Schill R. O. Mali B. Dandekar T. Schnolzer M. Reuter D. Frohme M. (2009). Molecular mechanisms of tolerance in tardigrades: new perspectives for preservation and stabilization of biological material. *Biotechnol. Adv.* 27 348–352. 10.1016/j.biotechadv.2009.01.011 19472511

[B98] Shukla E. Thorat L. Bendre A. Jadhav S. Pal J. K. Nath B. B. (2018). Cloning and characterization of trehalase: a conserved glycosidase from oriental midge, *Chironomus ramosus*. *3 Biotech* 8 352–358. 10.1007/s13205-018-1376-y 30105177 PMC6070458

[B99] Shukla E. Thorat L. Bhavnani V. Bendre A. Pal J. K. Nath B. B. (2016). Molecular cloning and in silico studies of physiologically significant trehalose from *Drosophila melanogaster*. *Int. J. Biol. Macromol.* 92 282–292. 10.1016/j.ijbiomac.2016.06.097 27377458

[B100] Shukla E. Thorat L. Nath B. B. Gaikwad S. M. (2015). Insect trehalase: physiological significance and potential applications. *Glycobiology* 25 357–367. 10.1093/glycob/cwu125 25429048

[B101] Silverman J. Rust M. K. (1983). Some abiotic factors affecting the survival of the cat flea, *Ctenocephalides felis* (Siphonaptera: Pulicidae). *Environ. Entomol.* 12 490–495. 10.1093/ee/12.2.490

[B102] Simelane D. O. (2007). Influence of temperature, photoperiod and humidity on oviposition and egg hatch of the root-feeding flea beetle *Longitarsus bethae* (Chrysomelidae: Alticinae), a natural enemy of the weed *Lantana camara* (Verbenaceae). *Bull. Entomol. Res.* 97 111–116. 10.1017/S0007485307004713 17411475

[B103] Sinclair B. J. Gibbs A. G. Roberts S. P. (2007). Gene transcription during exposure to, and recovery from, cold and desiccation stress in *Drosophila melanogaster*. *Insect Mol. Biol.* 16 435–443. 10.1111/j.1365-2583.2007.00739.x 17506850

[B104] Sjursen H. Bayley M. Holmstrup M. (2001). Enhanced drought tolerance of a soil-dwelling springtail by pre-acclimation to mild desiccation stress. *J. Insect Physiol.* 47 1021–1027. 10.1016/S0022-1910(01)00078-6 11472765

[B105] Sogame Y. Kikawada T. (2017). Current findings on the molecular mechanisms underlying anhydrobiosis in *Polypedilum vanderplanki*. *Curr. Opin. Insect Sci.* 19 16–21. 10.1016/j.cois.2016.10.008 28521938

[B106] Sota T. Mogi M. (1992). Interspecific variation in desiccation survival time of Aedes (stegomyia) mosquito eggs is correlated with habitat and egg size. *Oecologia* 90 353–358. 10.1007/BF00317691 28313521

[B107] Strachan S. R. Chester E. T. Robson B. J. (2015). Freshwater invertebrate life history strategies for surviving desiccation. *Springer Sci. Rev.* 3 57–75. 10.1007/s40362-015-0031-9

[B108] Suemoto T. Kawai K. Imabayashi H. A. (2004). Comparison of desiccation tolerance among 12 species of chironomid larvae. *Hydrobiologia* 515 107–114. 10.1023/B:HYDR.0000027322.11005.20

[B109] Tammariello S. P. Rinehart J. P. Denlinger D. L. (1999). Desiccation elicits heat shock protein transcription in the flesh fly, Sacrophaga crassipalpis, but does not enhance tolerance to high or low temperature. *J. Insect Physiol.* 45 933–938. 10.1016/S0022-1910(99)00073-6 12770286

[B110] Teets N. M. Peyton J. T. Colinet H. Renault D. Kelley J. L. Kawarasaki Y. (2012). Gene expression changes governing extreme dehydration tolerance in an Antarctic insect. *Proc. Natl. Acad. Sci. U.S.A.* 109 20744–20749. 10.1073/pnas.1218661109 23197828 PMC3528566

[B111] Terhzaz S. Teets N. M. Cabrero P. Henderson L. Ritchie M. G. Nachman R. J. (2015). Insect capa neuropeptides impact desiccation and cold tolerance. *Proc. Natl. Acad. Sci. U.S.A.* 112 2882–2887. 10.1073/pnas.1501518112 25730885 PMC4352776

[B112] Thorat J. T. Gaikwad S. M. Nath B. B. (2012). Trehalose as an indicator of desiccation stress in *Drosophila melanogaster* larvae: a potential marker of anhydrobiosis. *Biochem. Biophys. Res. Commun.* 419 638–642. 10.1016/j.bbrc.2012.02.065 22387478

[B113] Thorat L. Mani K. Thankgaraj P. Chatterjee S. Nath B. B. (2016a). Downregulation of dTps1 in *Drosophila melanogaster* larvae confirms involvement of trehalose in redox regulation following desiccation. *Cell Stress Chaperon.* 21 285–294. 10.1007/s12192-015-0658-0 26577464 PMC4786531

[B114] Thorat L. Nath B. B. (2015). Tolerance to desiccation stress in *Chironomus ramosus* through plasticity in homeostatic control. *Eur. J. Environ. Sci.* 5 86–91. 10.14712/23361964.2015.81

[B115] Thorat L. Nath B. B. (2016). Quantitative assessment of larval desiccation tolerance in oriental Chironomus species. *Curr. Sci.* 111 1448–1449.

[B116] Thorat L. Nath B. B. (2018). Aquatic silk proteins in Chironomus: a review. *J. Limnol.* 77 95–103. 10.4081/jlimnol.2018.1797

[B117] Thorat L. Oulkar D. Banerjee K. Gaikwad S. Nath B. B. (2017). High-throughput mass spectrometry analysis revealed a role for glucosamine in potentiating recovery following desiccation stress in Chironomus. *Sci. Rep.* 7 3659–3671. 10.1038/s41598-017-03572-5 28623254 PMC5473918

[B118] Thorat L. Oulkar D. Banerjee K. Nath B. B. (2016b). Desiccation stress induces developmental heterochrony in *Drosophila melanogaster* following desiccation stress. *J. Biosci.* 41 331–339. 27581925 10.1007/s12038-016-9628-7

[B119] Tichy H. J. (1979). Hygro- and thermoreceptive triad in antennal sensillum of the stick insect, *Carausius morosus*. *Comp. Physiol.* 132 149–152. 10.1007/BF00610718

[B120] Treherne J. E. Willmer P. G. (1975). Hormonal control of integumentary water loss: evidence for a novel neuroendocrine system in an insect (Periplaneta americana). *J. Exp. Biol.* 63 143–159. 1159358 10.1242/jeb.63.1.143

[B121] Tunnacliffe A. Lapinski J. (2003). Resurrecting Van Leeuwenhoek’s rotifers: a reappraisal of the role of disaccharides in anhydrobiosis. *Philos. Trans. R. Soc. Lond. B* 358 1755–1771. 10.1098/rstb.2002.1214 14561331 PMC1693263

[B122] Tunnacliffe A. Lapinski J. McGee B. A. (2005). Putative LEA protein, but no trehalose, is present in anhydrobiotic bdelloid rotifers. *Hydrobiologia* 546 315–321. 10.1007/1-4020-4408-9_32

[B123] Verlinden H. Vleugels R. Marchal E. Badisco L. Pflüger H.-J. Blenau W. (2010). The role of octopamine in locusts and other arthropods. *J. Insect Physiol.* 56 854–867. 10.1016/j.jinsphys.2010.05.018 20621695

[B124] Wadaka M. Mouline K. Parvy J.-P. Lannic J. L. Dabiré K. R. Ouédraogo G. A. (2016). Morphological changes in the spiracles of *Anopheles gambiae* s.l (Diptera) as a response to the dry season conditions in Burkina Faso (West Africa). *Parasit. Vectors* 69:11. 10.1186/s13071-015-1289-0 26739500 PMC4704408

[B125] Warren M. Robertson M. P. Greeff J. M. (2010). A comparative approach to understanding factors limiting abundance patterns and distributions in a fig tree-fig wasp mutualism. *Ecography* 33 148–158. 10.1111/j.1600-0587.2009.06041.x

[B126] Watanabe K. Imanishi S. Akiduki G. Cornette R. Okuda T. (2016). Air-dried cells from the anhydrobiotic insect, *Polypedilum vanderplanki*, can survive long term preservation at room temperature and retain proliferation potential after rehydration. *Cryobiology* 73 93–98. 10.1016/j.cryobiol.2016.05.006 27207249

[B127] Watanabe M. (2006). Anhydrobiosis in invertebrates. *App. Entomol. Zool.* 41 15–31. 10.1303/aez.2006.15

[B128] Weldon C. W. Boardman L. Marlin D. Terblanche J. S. (2016). Physiological mechanisms of dehydration tolerance contribute to the invasion potential of *Ceratitis capitata* (Wiedemann) (Diptera: Tephritidae) relative to its less widely distributed congeners. *Front. Zool.* 13:15. 10.1186/s12983-016-0147-z 27034703 PMC4815119

[B129] Wickson S. Chester E. T. Robson B. J. (2012). Aestivation provides flexible mechanisms for survival of stream drying in a larval trichopteran (Leptoceridae). *Mar. Freshw. Res.* 63 821–826. 10.1071/MF12095

[B130] Willmer P. G. (1980). The effects of a fluctuating environment on the water relations of larval Lepidoptera. *Ecol. Entomol.* 5 271–292. 10.1111/j.1365-2311.1980.tb01150.x

[B131] Xie Q. Zhang R. (2007). Responses of oriental fruit fly (diptera: tephritidae) third instars to desiccation and immersion. *J. Agric. Urban Entomol.* 24 1–11. 10.3954/1523-5475-24.1.1

[B132] Yoder J. A. Benoit J. B. Denlinger D. L. Rivers D. B. (2006). Stress-induced accumulation of glycerol in the flesh fly, *Sarcophaga bullata*: evidence indicating anti-desiccant and cryoprotectant functions of this polyol and a role for the brain in coordinating the response. *J. Insect Physiol.* 52 202–214. 10.1016/j.jinsphys.2005.10.005 16290823

[B133] Yoshida M. Matsuda H. Kubo H. Nishimura T. (2016). Molecular characterization of Tps1 and Treh genes in Drosophila and their role in body water homeostasis. *Sci. Rep.* 6:30582. 10.1038/srep30582 27469628 PMC4965777

[B134] Zukowski J. Su N.-Y. (2017). Survival of termites (isoptera) exposed to various levels of relative humidity (RH) and water availability and their RH preferences. *Fla. Entomol.* 100 532–538. 10.1653/024.100.0307

